# Environmental Materialities and the History of Pandemics

**DOI:** 10.1093/jhmas/jrae007

**Published:** 2024-05-23

**Authors:** Emily Webster

**Affiliations:** Durham University, UK

**Keywords:** environmental history, epidemics, materiality/neo-materialism, Anthropocene, ecology, history of disease

## Abstract

Over the last several decades, a growing group of environmental and medical historians have argued that engagement with the materiality of disease is critical to eroding the false boundaries between environment and health, and especially to the historical study of major epidemics and pandemics. This article evaluates the ways in which environmental and medical historians have engaged materiality when thinking through questions of infectious disease. It argues that far from eschewing cultural constructions of disease and analysis of medical systems, these works demonstrate that engagement with materiality in the study of disease articulates the stakes of medical regimes and practices of healing, and renders legible the multiple scales at which epidemics occur. Addressing key controversies in the use of sources, it provides examples of works that incorporate material objects, biological ideas and actors, and non-humans without falling prey to the extremes of “biological determinism” or “constructivism.” It argues that commonalities in the methods employed by these works – utilization of scientific frameworks and data, multispecies analysis, attention to scale, and spatial thinking – reveal unseen and untold aspects of past pandemics. It concludes with a brief example of how these frameworks come together in practice through a case study on the history of enteric fever in Dublin, Ireland.

## Introduction

The intwined languages of ecology, pathology, and epidemiology have proliferated daily conversation since the official declaration of the COVID-19 pandemic. From investigations into the initial outbreak and the virus’s origins, to catalogues of diverse symptoms and experiences around infection, scientific methods have shaped the questions we ask of disease, the places we look, and even how we conceptualize our individual experience of it. This is a familiar “framing,” to think about a disease in its intellectual, social, and scientific context.^1^ But as environmental historians have often noted, these observations also point to another, critical role that scientific thought plays in society: opening imaginative and analytic space to see facets of material and lived experience previously inaccessible to the historian.

The use of scientific frames in history has been controversial in social studies of medicine, often conjuring specters of “biological determinism.” Engagement with evolution and ecology, a defining practice in environmental history, has been central to these debates. In early works of environmental history, careless approaches to ecological and evolutionary ideas often led to deeply problematic interpretations. Alfred Crosby’s influential *Ecological Imperialism* (1986), and perhaps even more so *The Columbian Exchange* (1972), for example, have been critiqued by environmental historians for presenting a unidirectional thesis of biological imperialism that failed to consider how biological objects from the colonies in turn “colonized” Europe, or the historical context of how indigenous ecosystems and communities reacted to the introduction of new species.^2^ Others, such as William McNeill’s *Plagues and Peoples,* often reduced complex geo-political events such as the rise and fall of civilizations to the trajectory of a single pathological organism.^3^ Historians of medicine and the environment have viewed these works as complicit in biological determinism and the perpetuation of racialized interpretations of history.^4^ It is against these analyses that the cultural turn was built, and for several decades biology played a limited role in the historical analysis of epidemics.

In the intervening decades, however, the fields of evolution and ecology changed in ways that rendered the historical works of the 1960s and 1970s even less representative in their methodological and conceptual approaches. In the 1970s and 1980s, debates raged in evolution between scholars such as Richard Dawkins who embraced “sociobiology,” which explicitly linked biological fitness and genetic variability to individual and group-level success, and a much larger group of evolutionary ecologists, represented by Richard Lewontin and Richard Levin, who argued that biological reductionism is incompatible with ecology, claiming instead that separating “environment” broadly construed from the organism and its biology represents “an obstacle to further understanding.”^5^ In the 1990s, the conceptual framework of evolution and ecology shifted towards this latter perspective. The emergence of theories such as niche construction, epigenetics, inclusive inheritance, and other insights into pressures that influenced population and individual survival gave rise to a new, extended evolutionary synthesis (EES).^6^ Under this framework, the development of organisms and communities were thought to be influenced by a composite of “genetic, epigenetic, and ecological (including cultural) inheritance.”^7^ These shifts make quite clear that if biological reductionism persists in history, it is not because biology is itself reductive.

Theoretical expansions of ecology and evolution were simultaneously influenced by and shaped our understanding of how a new geological era, the Anthropocene, erodes the boundaries of what constitutes human health and medicine. As historian Tim LeCain notes, “we live in a time when our basic understanding of what it means to be ‘human’ is radically changing…the human body, mind, and culture are even more deeply embedded in our biological and material environments than we could have previously imagined.”^8^ This shift is perhaps most evident in the emergence of integrative public health methods, which have emphasized the importance of multi-scalar social, cultural, and economic practices in ecological change.^9^ Multidisciplinary health movements, including One Health, EcoHealth, and more recently Planetary Health, advocate “holistic,” more-than-human approaches to medicine and public health, arguing that human and environmental health must be considered inseparable.^10^

In line with these discoveries and shifting epistemologies, environmental and medical historians have argued that engagement with environmental materiality (here defined as the physical space and “things,” living and non-living, that comprise the environmental system) and specifically the environmental materiality of disease (pertaining to the things and physical space, including biological and ecological materials, that comprise the disease and its environment specifically) is critical to eroding the false boundaries between environment and health, and especially to the historical study of major epidemics and pandemics.^11^ These histories largely approach materiality from two, often intertwined, angles: engagement with materiality as a lens, drawing on numerous frameworks that facilitate materialist readings of historical disease events, including disease ecology, niche construction theory, and what we might broadly call “ecological Marxism” to frame their work; and those which ground historical evidence in forms of biological and ecological materiality, including engagement with ancient DNA (aDNA) techniques, climate data, and spatial and geographic data. Far from eschewing cultural constructions of disease and analysis of medical systems, these works demonstrate that engagement with the environmental materiality of disease articulates the stakes of medical regimes and practices of healing, rendering legible the multiple scales at which at which epidemics occur — factors which, I argue, are essential to understand a pandemic event such as COVID-19.

This essay explores how more actively addressing environmental materiality in the history of medicine and public health reveals new areas of interrogation and understanding, especially in the history of epidemics. Addressing key controversies in the use of sources, it provides examples of works that incorporate material objects, biological ideas and actors, and non-humans without falling prey to the extremes of biological determinism or constructivism. While by no means a comprehensive review of the literature, it draws on a few key examples to argue that the commonalities in the methods employed by these works — utilization of scientific frameworks, multispecies analysis, and attention to scale — and sources of data — including the use of biological and scientific material and spatial data —reveal unseen and untold aspects of past pandemics. Finally, I provide a brief example of how these frameworks come together in practice, and the new aspects of epidemic history that can arise from direct engagement with environmental materiality.

## Materiality as an Analytic

In a 2015 forum on technology, ecology, and human health since 1850, a formidable group of environmental historians assembled to think through the value of methodological engagement with “technological networks, ecological disruption, new evolutionary niches, novel materials, mismatch diseases, and knowledge production” in the Anthropocene.^12^ The unprecedented technological and ecological changes of the last two centuries, they argued, exposed the material specificity of disease to an unprecedented extent, emphasizing that “all diseases have their own pathogen-ecology system…composed of specific environments, disease agents, and bodies.”^13^ This observation was representative of a larger sea change in historical imaginations of environment and health. In their attempt to grapple with the epistemological challenges these realizations pose to the discipline of history, historians of disease and the environment have experimented with analytic frameworks for accessing this pathogen-ecology system and teasing out its implications. Together, three notable methods have emerged to reimagine human relationships to the material environment: critical engagement with ecological theories as framework; incorporation of animal and non-human materialities into histories of disease; and the conscientious use of scale to convey interrelationalities.

### Ecological Theories as Material Analytics

To write a history that appropriately considers the roles of ecology and human-non-human interaction in historical epidemics requires analytic frameworks that account for the life processes of all organisms and render visible their consequences for shared ecosystems. In the last two decades, disease ecology, public health, evolution and genetics have undergone methodological expansions, employing theories and practices that prioritize systems and networks of living organisms in their biological as well as cultural complexity. The policy recommendations of Johnathan Patz and co-authors on land use change and infectious disease, for example, show that factors implicated in cases of infectious disease are as widespread as biodiversity, pathogenicity, health care systems, disease-reduction practices, agriculture, and possibility of economic gain.^14^ These frameworks highlight the importance of both biological and cultural factors in influencing disease trajectory — and the presence of methodologies that might engage both critically. Looking to scientific frameworks in ecology and evolution that expose these interrelationships, I argue, provides important tools for writing sophisticated histories of disease.

The use of contemporary disease ecology to interrogate historical public health and environmental change is already commonplace in the history of disease. This methodology has been fruitful in studies of imperialism and epidemics, in which disease ecology is used to examine colonial disease management practices and their long-term implications.^15^ Scholars such as Chris Otter and Guenter Risse have engaged disease ecology to consider the emergence and success of pathogens on a global scale, examining how technology and environmental transformation intersect to “provide new niches for the recrudescence of older diseases and the emergence of novel ones.”^16^ In all of these works, evolution and ecology both provide the context for understanding the material consequences of technological and cultural change and are themselves historicized, a demarcation of concurrent knowledge formation and application.^17^

Meanwhile, some historians have embraced overtures by interdisciplinary fields to adopt and expand nascent methodologies that include both scientific and social-scientific frameworks. Niche construction theory, since its formal inauguration as a theory in 1993 by a group of evolutionary biologists, expanded to include ideas of cultural as well as biological niche construction, forming an analytically fruitful approach that addresses the dynamic material and biological effects of activities as wide-spanning as colonialism and capitalism and as specific as individual immunologic interactions to foreign pathogens.^18^ Historians have used niche construction to explore cases as widespread as the emergence of *Mycobacterium tuberculosis* in Australia, the global spread of the SARS epidemic, and the importance of yellow fever ecology to the outcome of imperial warfare.^19^ Other medical historians such as Warwick Anderson and James Dunk have seen value in novel frameworks such as planetary health for their “internal critique of colonialism and heedless economic growth,” using them for a radical epistemological reconfiguration of the boundaries of human health for historians and public health scholars alike.^20^

Beyond these explicit engagements, scientific methodologies and thought have also generated disease histories in more subtle ways, informing the lenses we use to think through human-non-human relationalities, logistics and supply, toxic chemical exposure and environmental health, and the movement of disease. Biographies of disease and cultural histories of epidemics, for example, often commence with a contemporary biological sketch, discussing its pathology, mortality rate, and epidemiology.^21^ Histories that examine the social determinants of health draw on and complement changing frameworks within the field of public health used to describe inequalities in disease morbidity and mortality. And perhaps more than ever, historians who have lived through COVID-19 are acutely aware of the ways that the biological changes to an organism dramatically affect and are affected by the physical manifestations of these disparities. Reflecting on these methods, we might ask, as historian Monica Green does, if dismissal of scientific methodologies as “retrospective diagnosis” have instead “become a major obstacle to fruitful dialogue between the humanistic and scientific approaches,” and even more so, if lack of engagement with scientific approaches may lead to unnecessarily limited interpretations of epidemics.^22^

### Multispecies Materialities

Less controversial in the history of disease is acknowledgement of the powerful role of multispecies materialities in epidemics. The majority of emerging and re-emerging infectious diseases with pandemic potential, both past and present, have been “spillover” diseases, or diseases which follow multispecies pathways.^23^ The specific ecologies of infectious disease emergence, once considered the stuff of specialist knowledge, have entered the mainstream in the search for the origins of SARS-CoV-2.^24^ Reminiscent of the SARS outbreak of 2002-2003, “wild” animals (sometimes captured, sometimes farmed) came under intense scrutiny as potential furry patient zeroes and —– as anthropologist Christos Lynteris pointed out early in the epidemic — the markets in which they were sold were exoticized and demonized.^25^ However, this is still merely one way that non-humans have played a central role in the story of COVID-19. From RNA vaccines to macaques as test subjects, from comfort pets to dying herds of deer, the story of COVID-19 is necessarily a more-than-human story.^26^

Whether we label them commensal or parasitic, companion or vector, animal-human relationalities drive the transformation of landscapes, the creation of technologies and artistic objects — and the emergence of disease. Under neo-materialist frameworks, historians have elevated non-humans to co-participants in history — noting that their particular biological, behavioral, and cultural features are as central to key historical events as human characteristics.^27^ In studies of epidemic disease, animals’ agency and historical consequentialism have been tied to public health, and their materiality understood as a challenge to the structure of human-centered or indeed, European-centered health systems.^28^

Vector-borne illnesses constitute a rich area of study because of their clear ecological origin, both retrospectively and compared to contemporaries. Histories of vector-borne illnesses draw explicit connections among colonial land use change, warfare, migration, inequality, race, and infrastructure in their spread. Among this formidable historiography, studies that have explicitly engaged a multispecies frame have often powerfully evaluated scientific and military regimes. From Daniel Headrick’s analysis of sleeping sickness campaigns as instigating an ecological crisis of imperialism through its effect on the tsetse fly to Dawn Biehler’s analysis of the relationship between racial geography and vector-borne illness, multispecies histories of disease have made explicit the political ecologies of epidemics, the scales of material environmental change implicated in disease, and the complex feedback loops between human and non-human activities that produce and sustain epidemics.^29^ Other scholarship uses animal materiality to form epistemic inquiries through how animals shape epidemic knowledge formation and influence the framing of animals in epidemics.^30^ Using plague once more as a model disease, Lynteris’s work considers how rats and other animals became “epidemic villains,” and equally importantly, how “the image and social life of non-humans as epidemic villains is a constitute part of public health as apparatuses of state and capitalist management.”^31^

An ecological framing of the role of non-humans in epidemics can also help us to see spaces and actors not traditionally implicated in epidemic ecologies. For example, work at the nexus of public health and urban environmental history gives epidemiological significance to a host of organisms moving through these less explicitly natural spaces. The constant buzzing of flies turns ominous when implicated in seasonal epidemics of salmonella, as Anne Hardy explores; meanwhile, the benign shellfish that dot the shores of a major European port city become deadly when a sewage pipe emptied over them.^32^ While non-humans feature in histories of knowledge networks and public health, they are rarely examined using an ecological frame; the individual organism carrying the disease is more often severed from the ecology that perpetuates it. While some works are beginning to bridge this gap, ecological public health histories remain an area of needed study.^33^ Indeed, until the ecological frames so prevalent in histories of colonial or tropical medicine are turned to the crowd diseases of Europe and the Americas prevalent in urban spaces, historians of disease risk perpetuating the exoticization at the heart of tropical and colonial medicine.

The aspects of COVID-19 that have captured public and expert imagination make clear there are still many roles for non-humans in historical time that have yet to be explored. Turning once again to the idea of incorporating scientific frames and forms of evidence in historical storytelling, we might, as a growing group of historians have highlighted, think about how recent discoveries in the microbiome erode the human-nonhuman divide altogether, showing that many non-humans are actually directly involved in the composition of human.^34^ What would histories that seek to incorporate these microbes look like? Histories of environment and medicine that engage animal materialities are well-placed to undo the obfuscation of Anthropocene infrastructures – to remind people that some of the most prolific and effective antibiotics were produced by microbes against one another and simply harnessed by humans; that ecological disruption and agricultural supply chains go hand-in-hand; and that the compartmentalization of the human body that dominates modern medical thinking is historically contingent — in short, to remind readers that health is, and always has been, a multispecies system.

### Scale as a Material Analytic

“Scale,” as Julia Adeney-Thomas has argued, “matters in history as well as biology.”^35^ The practice of history is synonymous with the practice of scaling – the decision by the individual, subfield, and field of historians to define the boundaries of an event or phenomenon “determines much about our understanding of it.”^36^ Historians of disease often employ scalar thinking in the conceptualization of the human-institution-health nexus to interrogate epidemics at the level of the neighborhood, city, or state. They also use scale to tell broad histories of disease that remain grounded in regional source materials, though thoughtful engagement with scale has only recently become a topic of discussion in the history of environment and disease.^37^ Notable exceptions to these traditions exist, especially when the biology of the organism and the material conditions of its spread lend itself to readings using differing scales – histories of the three plague pandemics, for example, are often told through the multi-scalar lenses of commerce, imperialism, and technology.^38^

However, the COVID-19 pandemic has served as a visceral reminder that the scales on which epidemics and pandemics operate are often far more diverse and complex than the scales in which we often choose to tell these stories. Emergence, under current operating theory, resulted from the encroachment of farmed exotic animals on forest ecologies, a function of land-use change and demand from a wealthy class seeking novelty; the patterns of morbidity and mortality of different viral strains were reliant on increased air travel, local public health policies, and topographies of privilege and vulnerability particular to time and place; and as many historians and epidemiologists alike have warned, the spread of this microscopic virus is linked to a planetary phenomenon constructed by global capitalism, and likely to be just one of many in the Anthropocene.^39^ To tell stories in these registers is a historical and ethical obligation, and one that requires a more nuanced and thoughtful engagement with scale.

Self-described materialist frames have made a practice of thinking with scale – as visible in histories of capitalism and environment. Scale is a foundational feature of economic materialism, and when employed by historians of capitalism to follow supply chains, reveals the alienation of persons from the means of production and its raw materials, or in ecological Marxism, the alienation of persons from nature.^40^ As Gregg Mitman has noted, employing differing scales of analysis can reveal obscured forms of capitalist violence in the study of pandemics, and in doing so, allows us to critically reflect on narratives of emergence we have come to accept.^41^ Pandemics, from a planetary perspective, are “foremost, products of capitalism as an ecological regime: eruptions flowing from deep fissures in nature-society relations, produced by capitalism’s insatiable appetite for cheap land and labour, which lay bare what the feminist science studies scholar Michelle Murphy calls the economization of life.”^42^

While sympathetic to the importance of capitalism as a multi-scalar analytic, other works suggest that we must also look to the overtly biological to understand scale, to “the history of life on this planet, the way different life-forms connect to one another, and the way the mass extinction of one species could spell danger for another.”^43^ Building on the work of Dipesh Chakrabarty, Anna Tsing, Michael Vann, and others, we must also think of the biological materiality of ecosystems – both human and non-human – as inherently multi-scalar and think through how systems that scale – from imperial networks to Anthropocene infrastructures – interact with this biological materiality to produce epidemics.^44^

Despite the popularity of these ground-breaking studies, epidemic histories that thoughtfully engage in multi-scalar analysis of materiality remain somewhat marginal. And yet, as the multiple, genetically, epidemiologically, and culturally distinct waves of the COVID-19 pandemic make clear, the complexity of epidemic disease is only truly visible when we examine events at appropriate biological and cultural scales. In addition to revealing obfuscated aspects of epidemic disease emergence, embracing scale may provide opportunities for historians of disease to open new conceptual spaces in interdisciplinary disease studies. Multiple scales of analysis are often difficult to convey for scientists tasked with explaining emergence. Following Linda Nash, we might consider that “contemporary epidemiologic methods do not handle social and structural variables all that well, nor is [epidemiology] well-suited to spanning the relevant scales of analysis” – that is, considering factors that span from local to global and are deeply embedded in cultural, historical, and socio-ecological contexts that do not generalize well nor are easily quantified. Thus, “[d]espite their statistical sophistication, cross-sectional and longitudinal studies may be less informative than careful historical work in revealing the multiple and overlapping causes behind shifts in…disease.”^45^ In short, there is important work to be done that historians are uniquely suited to perform, but which require that we once again engage materialist analytics, especially careful scaling, alongside our strong sociocultural studies of disease to tell more nuanced and important histories.

## Materiality as Archive

COVID-19, like many pandemics before it, has been overwhelmingly represented and understood spatially. Throughout the pandemic, millions surveilled the Johns Hopkins University COVID-19 counter, watching as countries became saturated with dark red as cases and deaths mounted and cities were surrounded by ever larger bubbles to denote their own, smaller epidemics.^46^ Neighborhood and district maps within the United States gave striking visual confirmations of the toll of inequality, as mortality and infection rates soared among people forced to leave their homes for work, take public transit, or otherwise live within infrastructures poorly suited to self-protection.^47^ Among experts, spatial analysis has been used to identify patterns of emergence where outbreak investigation has stalled, as a recent *Nature* article powerfully demonstrates.^48^ One cannot help but think of how historians of the future will be faced with millions of spatial representations of the COVID-19 pandemic, stored as maps or spatially-coded tables, with which to make meaning of the epidemic.

These spatial data points, combined with genetic surveillance, have also rendered visible another critical feature of the epidemic: the emergence and circulation of different strains. From our embodied experience and these surveillance practices, we acknowledge distinct COVID-19 variants as historically important; morbidity and mortality rates, patterns of infection globally and locally, vaccine effectiveness, and embodied experiences are in part reliant on the insertions, deletions, and point mutations that allow a new strain to emerge.^49^ These emergence events are themselves spatial, often driven by the evolutionary pressures of highly specific environments, both within and outside human hosts.

As these familiar examples demonstrate, it is not enough to think of materiality as a frame through which to access and analyze historical disease events; we must also think of the related but distinct process of engaging materiality as a *primary source*. While historians have long examined spatial data as a primary source, novel methods for extracting, analyzing, and visualizing spatial data invite new modes of engagement. Similarly, developments in microbiology, genetics, and ecology have created new sources of historical data. Incorporating these new types of data – from reconstructed and mapped lineages of DNA, to climate proxy data, to coded geographic data — historians might access past materialities in historiographically significant ways.

### Scientific Data as Historical Evidence

Genetics research and technology have opened new avenues through which to interrogate and ultimately settle major historiographic debates in pandemic history, and opened new avenues for engagement and critique. In no subfield is this more apparent than plague studies. Through engagement with the methods and results of aDNA analysis, for example, Monica Green argues that the microbe responsible for both the Black Death and the plague of Justinian has been definitely identified as *Yersinia pestis*, and that this identification “opens up entirely new questions, ones we did not previously know we needed to ask, and equally importantly, new ways of asking them.”^50^ Incorporating genetic and evolutionary evidence provides opportunities for historians to tell stories at different temporal and spatial scales. It also provides new analytic angles within existing narratives. Christian McMillen and Helen Bynum, for example, have recently utilized genetic and ecological sources to tell new histories of an ancient disease: tuberculosis.^51^ To Bynum, the physiology of *Mycobacterium tuberculosis* both guides its relationship with the body and plays an undeniable role in the medical practices levied to cure it for millennia.^52^ For McMillen, an understanding of the evolutionary history of the bacteria becomes a critical source for anti-racist histories, one that lays to rest (and appropriately historicizes) “Virgin Soil Epidemics.”^53^ In keeping with this theme, Claas Kirchhelle has argued that microbiological evidence can also reveal new facets of global medical regimes, open new avenues of inquiry about the biopolitics of vaccines, and expose epidemiologically consequential “hidden infrastructures.”^54^

Beyond data on the microbe itself, some historians of disease have begun to grapple with proxy data developed by climate scientists to examine possible links between climate variability and epidemic disease. While early works in this field fell prey to similar generalizing tendencies that plagued early histories of disease and environment, recent scholarship has advocated a more nuanced use of climate data.^55^ At the time of writing, only a handful of scholars have attempted to write disease histories that incorporate climatological proxy data, and significant challenges arise in the incomplete nature of the data and the scale of reference. As J. Luterbacher and coauthors note, often the temporal and spatial scales of human and climatological evidence do not align in productive ways.^56^ However, there is still room for more nuanced examinations of climate-disease relationships. Anthropocene scholars and historians of capitalism, for example, might find fertile ground in the examination of structures underlying post-1850 climatological shifts and the emergence and re-emergence of infectious disease.^57^ Indeed, historians of disease may be uniquely positioned to demonstrate responsible use of climatological sources, and to further nuance and contextualize conversations in public health linking climate and disease.

### Spatiality and Materiality

The pervasiveness of spatial analysis in epidemiology and outbreak investigation has ensured that engagement with spatiality and spatial sources is a critical component in historical studies of disease across subfields.^58^ Maps, as highly visual, layered representations of spatial data, are the most common point of spatial engagement for historians of disease, as well as one of the most common forms of spatial argument. As Tom Koch puts simply, “epidemics and pandemics are spatial phenomena; mapping them is how the public nature of a disease threat is seen and studied.”^59^ Historians of science and medicine have contended that maps are “living” objects, conducive to both analysis and meta-analysis in their form and content.^60^ Maps are not only ways of organizing knowledge into analytic forms and structures, but also assertions of value – what is and is not included in a map, and how space and time are conveyed, inextricable from their stated subject.

Historians of disease maps have demonstrated the myriad of ways that a disease map intersects with environment, person, community, political and economic boundaries, and forms an argument about the world.^61^ As Koch notes, mapping “uniquely encourages comparisons between the variety of disease experiences and the environmental realities that may promote or inhibit its incidence or spread.”^62^ A map is simultaneously subject and object, a conveyance of materiality as interpreted by the mapper in time and place. These observations are critical to both understanding how to approach disease maps and to teasing out the consequences of their creation.

In addition to the maps created by historical actors that serve as sources, the spatial data generated *about* the past is equally rich and under-engaged as a historical source in the study of disease. Climate models, archaeological reconstructions, spatial analyses of genetic material, and hydrology and elevation data, for example, all present differing models and representations of historical time. While subject to their own limitations, these sources present alternative data points for examining historical events, and in their contradictions or consistencies with other historical evidence, produce new questions and ways of seeing these events that are rich and historically productive. Jacques Pepin, Monica Green, and Stuart Borsch, for example, interweave historical maps with contemporary maps (both genetic and geographic) to delineate the role of infrastructural development, trans-Oceanic trade, and environmental change in the spread of major pandemics of HIV and plague, highlighting the spread of disease as an environmental, biological, medical, and colonial phenomenon.^63^ In each of these works, engagement with other forms of spatial materiality illuminates new historiographic arguments, lending new significance to existing historical sources.

While maps constitute the most obvious form of spatial data, environmental materialities that comprise space are also often conveyed in flattened, statistical forms, including nested tables, graphs, and charts. This may seem overly obvious; much has also been written about these historical objects, especially their use in organizational bureaucracies in the seventeenth, eighteenth, and nineteenth centuries.^64^ In public health practice, as Michel Foucault infamously noted, these descriptive datasets form the professional backbone of outbreak investigation, policy, and biopolitical control.^65^ However, these sources are often engaged with in their form of presentation, and rarely processed or visualized to render the data multi-dimensional. Learning how to engage this data in multiple forms, far from a specialty or niche skill, can help historians identify systematic biases and inconsistencies in data — and form a clearer picture of what the data actually reflects. While their limitations should be kept in mind — a challenge made easier by the extensive literature in the history of medicine that provides critical readings of mapping practices – tools such as ArcGIS and other spatial analytic software can help historians engage more rigorously with the spatial data present in historical sources and access material environments outside the confines of traditional archival sources. Treating spatial data as a source – and spatial data banks as archives – provides historians with greater analytic opportunities, allowing us to see the boundaries of the map as a “thinking device and a means of experimental combination,” more clearly and consider more carefully the ways that we choose to employ these sources and structures to tell stories of disease.^66^

Finally, we might consider how engaging with material environments through a variety of spatial data allows historians to critically interrogate the arguments that comprise maps and disassemble colonial lenses. As Lukas Engelmann suggests, statistics, maps, and other resources that produce and visualize quantitative material during the colonial period were often tools of oppression, used as instruments of “surveillance, social control and hygienic principle.”^67^ Perhaps this is where drawing spatial data from multiple sources has its greatest value: it allows us to read against the grain, introducing frames of analysis that incorporate ecological agency and textured socioecological relationships. By reading into the multiple layers of materiality presented in the map as well as its subjectivities, we can turn the colonial gaze on its head, seeing instead in these maps moments of power-driven ecological tension, minute relationships, and ways of living and being that themselves defy the hygienic gaze. Building a history of disease that incorporates nuanced contemporary biological and ecological knowledge in its analysis and use of space can, perhaps counterintuitively, be an act of resistance against reductionist and deterministic narratives of disease. Undertaken carefully, this lens of analysis provides an opportunity to examine these sources without repeating and being complicit in the biopolitical gaze that formed them.

## Case Study: The Persistence of Typhoid Fever in Dublin, Ireland

In the final section, I draw on a brief example from the collaborative project “Typhoid, Cockles, and Terrorism” to demonstrate how a study grounded in environmental materiality can reveal new angles on a classic disease.^68^

In 1880, Dr. Charles Cameron was nearly laughed out of a meeting of the British Medical Association. Drawing on extensive outbreak investigations he undertook as Medical Officer of Health for the city of Dublin, Cameron argued that shellfish were likely an important factor in typhoid transmission.^69^ While shellfish-borne typhoid would be legitimized as a public health threat across Europe near the turn of the century, Cameron’s observation predated official acceptance by over two decades. Instead, his research was met with incredulity and derision —the president of the association himself asked Cameron if this was “one of his Irish jokes.”^70^

By the time of Cameron’s report, a professional class of medical officers had acknowledged the likelihood of typhoid transmission by fecal contamination of water sources.^71^ However in Dublin, innovations in water infrastructure, including protection of reservoirs, sand filtration systems, and the introduction of water-carriage systems for sewage removal, had allowed for a “nearly perfect” water supply.^72^ Yet the problem that Cameron observed – that the city of Dublin continued to struggle with abnormally high typhoid fever mortality despite general trends towards declining mortality across the British Isles – persisted for nearly twenty-five years. In fact the epidemics worsened, resulting in hundreds of deaths and thousands of cases annually by the twentieth century, which medical officers continued to attribute to the “filthy habits amongst large classes of people.”^73^

To understand both why Dublin struggled with local epidemics during a period of general decline in typhoid mortality, and how Cameron observed the connection between typhoid and shellfish decades earlier than his professional associates, requires seeing Dublin’s typhoid epidemic as grounded in a particular material environment, and engaging a few key aspects of environmental materiality: the biology of *Salmonella enterica* serovar Typhi, the multiple scales of pressure enacted on the environment by imperial economies and local practices, and the spatial geography of the disease as reconstructed through vital statistics and historical ecological data. Together, these elements reveal a unique epidemiology of typhoid fever that directly challenged contemporary epidemiological approaches.

Dublin, as the commercial and administrative seat of British-occupied Ireland, was a site of particular attention for sanitary improvement and modernization projects at the end of the nineteenth century. It was also a vibrant and historic Irish port city, home to unique socio-ecological relationships, the quotidian rhythms of which often ran counter to the imposed ecologies of improvement. It was this tension, I argue, that provided a unique niche for the city’s typhoid fever epidemics.

Typhoid fever is a human-specific, fecal-oral infection. Transmission of its causative bacteria, *Salmonella enterica* serovar Typhi, can occur via what epidemiologists refer to as “short-cycle” or “long-cycle” transmission. Short-cycle transmission includes the common risk factors associated with typhoid, such as a lack of handwashing and poor home-based sanitation, or using pit latrines instead of water closets.^74^ Long-cycle transmission, on the other hand, occurs through environmental mediators – indirect transmission by contaminated water, sewage outfalls, milk, or even soil. At the end of the nineteenth century, as Jacob-Steere Williams has argued, long-cycle transmission municipal factors such as water contamination or food regulation were often targeted for intervention, but often outbreaks were attributed to a single person or group of people’s perceived inability to maintain hygienic standards (short cycle).^75^

However, planetary health studies of typhoid suggest that another scale of transmission is consequential – what might be called extra-long cycle factors. These studies argue for the importance of landscape morphology, presence of watershed or catchment areas, river characteristics, and other upstream drivers of health.^76^ Environmental epidemiologists have found that the centralization of water drainage into a single large, meandering river in sub-catchment ecologies allowed pollutants to accumulate with water runoff, which increased nutrient concentrations, which in turn fostered the growth of *Salmonella typhi* and fecal coliform bacteria.^77^

Drawing on the ecological theory of niche construction to consider environmental factors that could influence local typhoid transmission, some topographical and hydrological features appear relevant. The position of Dublin at the lowest elevation in a sub-catchment basin, the composition of the city’s soils, and its high average rainfall suggest the city may support a number of these extra-long cycle risk factors for typhoid, providing an ideal ecological niche for the bacteria. These risk factors form only part of the story, however; as analysis of medical officer of health reports, government inquiries, and geography revealed, typhoid incidence reflected how humans and non-humans living within that basin interacted with it and each other. Cockles and other bivalve shellfish thrive in estuarial ecologies, which offer protection from the strong tides of the ocean and soft, silty mud in which to burrow. These organisms are filter-feeders, taking in and incorporating nutrients and other particles. They are, for this reason, often considered to be a canary in the coal mine of water pollution – and potential harbors of infectious bacteria.^78^

The significance of this ecology becomes clear when it is embedded in its historical cultural context. The practice of cockle-gathering has a rich history along the Irish coast. Shellfish collection was often a leisure activity, or an economical supplement to an otherwise uncolorful diet among Irish working classes. In his report on the Sanitary State of Dublin in 1906, Surgeon-Colonel D. Edgar Flinn, a medical inspector for the Local Government Board of Ireland, noted that cockle-gathering and hawking were both widespread, especially in summer and autumn months — when typhoid fever rates reached a notable peak.^79^ Flinn noted, “The cheapness of cockles as an article of dietary brings them within the reach of the poorer classes, hence their consumption is considerable, and a large number of people gather them in addition to those who earn a livelihood by selling them.”^80^ Cockle consumption was a quotidian activity for the working class and poor of the city – a socioecological relationship forged by the sub-catchment basin and its organisms. In other words, the materiality of cockles as animals and their socio-ecological relationships with humans directly affected human disease risk.

A growing population meant that a large number of people supplemented their diet with Dublin Bay’s cockles at the time of Cameron’s observations. Dublin had grown enormously in the late nineteenth century, as people migrated to the city from the countryside in search of work, and simultaneously, imperial administrators targeted it for improvement. High death rates for the city, often tied to its low-lying, waterlogged geography, provided the impetus for the development of a Main Drainage Scheme. By the late 1870s, a Royal Commission called by the British government to address the city’s unsanitary environs selected a scheme developed by Park Neville, in consultation with Sir Joseph Bazalgette, the chief engineer for the London main drainage scheme.^81^ Under the scheme, a series of smaller sewers around the city connected to a large arterial drain, which emptied near Pigeon Fort into Dublin Bay.

The proposed plan seeded tensions between the city and the state; local engineers cautioned that the areas most used for cockle gathering would be inundated with sewage. John Purser Griffith, Assistant Engineer to the Port and Docks Board, noted that while “the city would be greatly benefited if the sewage were discharged at the proposed outlet…the district of Clontarf would suffer very materially from sewage washed upon the strand,” citing the tidal patterns and the slow velocity of the current within the Bay.^82^ This advice was largely ignored in favor of sanitary advice from the metropole, however, and construction proceeded from the 1880s onward.^83^ Understanding the sanitary system as a result of multiple scales of political tensions that prioritized forms of knowledge from the metropole over those of local engineers provides insight into the somewhat predictable result: the maladaptation of the sanitary system to the city’s needs.

**Figure 1.**
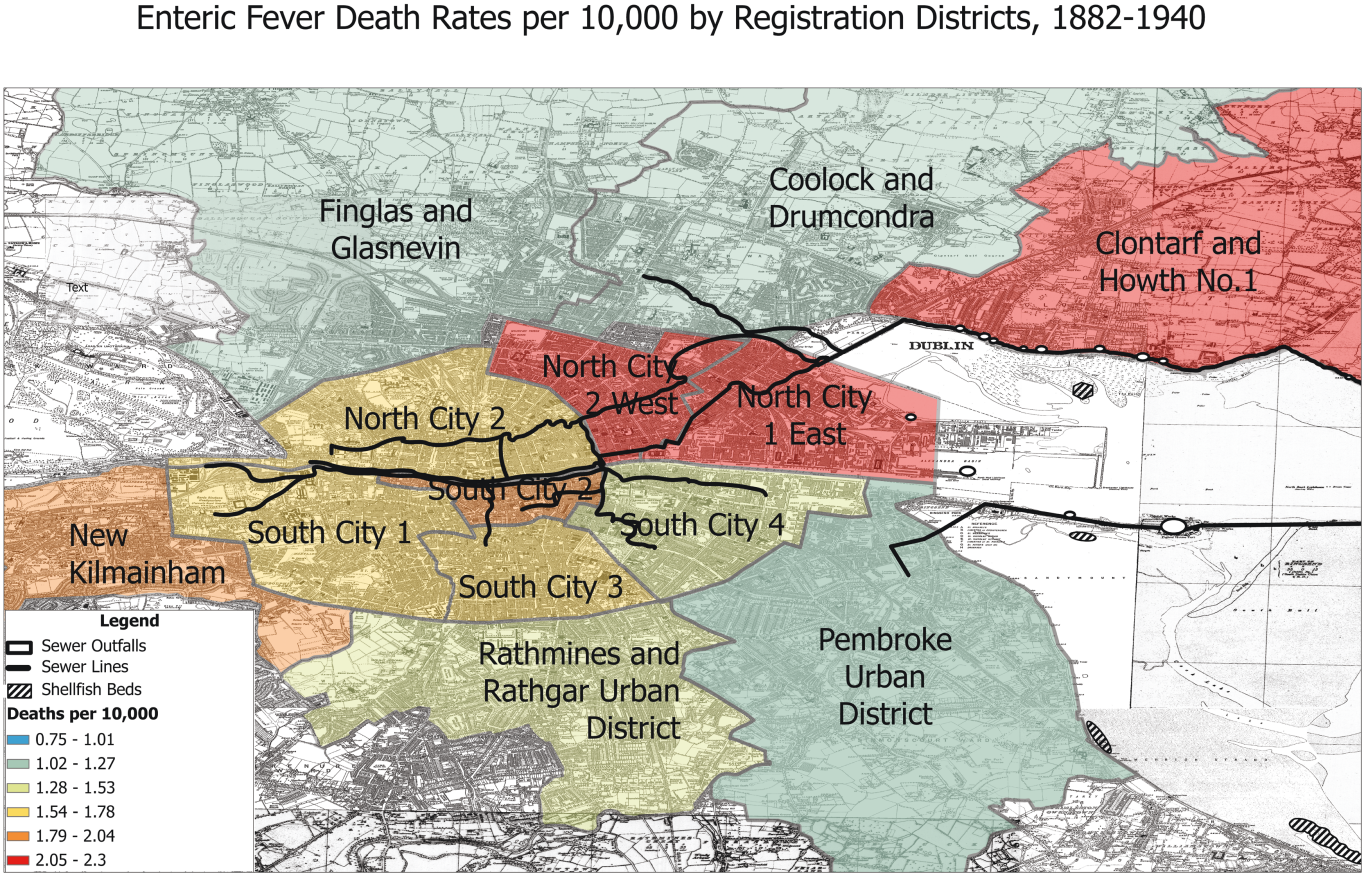
Enteric Fever Death Rates per 10,000 by Registration District, 1882-1940. SOURCE: © OpenStreetMap Contributors. Base Map Ordinance Survey of Ireland, 1937. Mortality data compiled from Charles Cameron, Medical Officer of Health Reports, 1881-1941.

Visualizations of registrar’s and vitality statistics collected annually by the chief medical officer of health in the period following the construction of the main drainage scheme alongside the lines of the sewers proposed suggests that local experts’ concerns were realized: looking at cumulative mortality rates by neighborhood between 1882-1940, we can see that the highest death rates were concentrated in Clontarf and Howth, in the north, and along the North side of the city – near some of the city’s largest shellfish beds (Figure 1). Occupational mortality statistics provide further evidence for the connection between shellfish gathering and typhoid. In 1888, Cameron, who had backed off from his initial stance on the relationship between cockles and typhoid fever but continued to investigate its causes, noted that of 168 deaths from typhoid that year, 68 of them (or forty percent) occurred among those classed as “Hawkers, Porters, Labourers, &c.”^84^ By visualizing this statistical data spatially and alongside infrastructural and environmental data, patterns in epidemiology become visible that may have otherwise remained obscured.

As evidence for the relationship between typhoid fever and shellfish consumption mounted both locally and in British professional circles, medical officers of health across Ireland, Cameron included, began public awareness campaigns to discourage the practice among the city’s residents. The first pamphlets warning of typhoid-infected shellfish circulated in Dublin in 1903.^85^ Targeted reports and testimonies supplied by Cameron to the Port and Docks Board and the Local Board of Health also led to the first closure of oyster layings in the United Kingdom on sanitary grounds.^86^ While typhoid fever rates gradually declined over the twentieth century, newspaper articles tracing typhoid infections to local cockle gathering practices continued well after the Second World War – highlighting the robustness of Dublin’s ecology as a niche for typhoid fever.^87^

As this brief example demonstrates, engagement with some of the major themes outlined in this paper can open new avenues of inquiry in the study of epidemics and bring novel interpretations to historical sources. By engaging with the disease ecology of *Salmonella enterica* serovar Typhi, the socio-ecology of human-nonhuman relationships, the multiple scales of urban public health infrastructure, and visualizing mortality statistics and geographic data, a more textured narrative of typhoid mortality in Dublin is visible. While it is impossible to say what portion of typhoid infections arose from the consumption of infected shellfish, analysis through this ecological and material lens allows for a more nuanced history of typhoid in Dublin that counters imperial narratives often used to explain the city’s high infectious disease rates. Far from a disease of filth and overcrowding, as most historiographies still suggest, the typhoid fever epidemic that raged across the city arose from a clash of local ecologies and imperial sanitary infrastructures.

## Conclusion: Towards A Mindful Materialism

Much as the COVID-19 pandemic has rendered clear the collapse of the age-old nature-culture distinction, it has also made more explicit than ever the need for more robust engagement with materiality in the history of disease. Examining the many nuanced works of history that have engaged environmental materiality through various methodological lenses in the last decade, it becomes clear that materialism can no longer be considered overly simplistic or reductionist in its approach to history. Confronting the existential challenges to health implicit in the Anthropocene, from anti-microbial resistance to emerging infectious diseases to microbiome studies, our engagement with medical and scientific knowledge and practices must transcend dismissal of methods as “retrospective diagnosis,” instead asking the more pressing question of how they allow us to see our sources, actors, and past environments anew. However, to avoid the specter of reductionism, we must also learn from these past disciplinary fissures, and continue to “subject scientific work to the same historicizing process” as other frames and sources.^88^ Undertaken mindfully, works in this field can provide a crucial example of how to evaluate scientific methodologies critically while still employing them to understand and reveal new aspects of historical epidemics – a skill that today seems more pressing and necessary than ever.

## Footnotes

1 Charles E. Rosenberg and Janet Lynne Golden, ed., *Framing Disease: Studies in Cultural History* (New Brunswick, NJ: Rutgers University Press, 1992).

2 Linda Nash, “Beyond Virgin Soil Epidemics,” in *The Oxford Handbook of Environmental History*, ed. Andrew C. Isenberg (Oxford: Oxford University Press, 2014); David S. Jones, “Virgin Soil Epidemics,” *William and Mary Quarterly* 60 (2003): 703-742; Alfred W. Crosby, *Ecological Imperialism: The Biological Expansion of Europe, 900-1900*, 2^nd^ ed. (Cambridge: Cambridge University Press, 2004); Alfred W. Crosby, *The Columbian Exchange: Biological and Cultural Consequences of 1492*, 30^th^ anniv. ed. (Westport, CT: Praeger, 2003).

3 William Hardy McNeill, *Plagues and Peoples* (New York: Anchor Books, 1998 [1976]); Jared M. Diamond, *Guns, Germs, and Steel: The Fates of Human Societies* (New York: Norton, 2005).

4 Nash, “Beyond Virgin Soil Epidemics,” 80. David Jones, on the other hand, argues that Crosby actually downplayed the genetic weakness hypothesis and instead emphasized many environmental factors, but argues that subsequent historical work needs to be extremely nuanced in its treatment of the social determinants of disease or risk being interpreted as supporting racial theories. See Jones, “Virgin Soil Epidemics.”

5 Richard Levins and Richard Charles Lewontin, *The Dialectical Biologist* (Cambridge, MA: Harvard University Press, 1987), 50-58; Richard Levins, “Is Capitalism a Disease?” *Monthly Review*, 1 September 2000, https://monthlyreview.org/2000/09/01/is-capitalism-a-disease/.

6 Massimo Pigliucci and Gerd Müller, ed., *Evolution, the Extended Synthesis* (Cambridge, MA: MIT Press, 2010); Gerd B. Müller, “Why an Extended Evolutionary Synthesis is Necessary,” *Interface Focus* 7 (2015): 20170015.

7 Kevin N. Laland, Tobias Uller, Marcus W. Feldman, Kim Sterelny, Gerd B. Müller, Armin Moczek, Eva Jablonka, John Odling-Smee, “The Extended Evolutionary Synthesis: Its Structure, Assumptions and Predictions,” *Proceedings of the Royal Society B: Biological Sciences* 282 (2015): 20151019.

8 Timothy J. LeCain, *The Matter of History: How Things Create the Past* (Cambridge: Cambridge University Press, 2017), 21-22.

9 Sarah Whitmee, Andy Haines, Chris Beyrer, Frederick Boltz, Anthony G. Capon, Braulio Ferreira de Souza Dias, Alex Ezeh, Howard Frumkin, Peng Gong, Peter Head, Richard Horton, Georgina M. Mace, Robert Marten, Samuel S. Myers, Sania Nishtar, Steven A. Osofsky, Subhrendu K. Pattanayak, Montira J. Pongsiri, Cristina Romanelli, Agnes Soucat, Jeanette Vega, Derek Yach, “Safeguarding Human Health in the Anthropocene Epoch: Report of The Rockefeller Foundation–Lancet Commission on Planetary Health,” *Lancet* 386 (2015): 1973–2028.

10 For a review of these approaches, see Henrik Lerner and Charlotte Berg, “A Comparison of Three Holistic Approaches to Health: One Health, EcoHealth, and Planetary Health,” *Frontiers in Veterinary Science* 4 (2017): 163; James Dunk and Warwick Anderson, “Assembling Planetary Health: Histories of the Future,” in *Planetary Health: Protecting Nature to Protect Ourselves,* ed. Samuel Myers and Howard Frumkin (Washington, DC: Island Press, 2020), 26-29. Dunk and Anderson trace the origins of planetary health to Anthony McMichael and Andy Haines, who united a planetary frame, systems ecology, and epidemiology in the 1990s. It has since grown considerably into an international, multidisciplinary health movement.

11 Materiality as comprised of the “things” that form a broader environment and field of interaction is a concept delineated by political theorist Jane Bennett, and offers the closest definition to what this body of work seeks to articulate. See Jane Bennett, *Vibrant Matter: A Political Ecology of Things* (Durham: Duke University Press, 2010).

12 Chris Otter, Nicholas Breyfogle, John L. Brooke, Mari K. Webel, Matthew Klingle, Andrew Price-Smith, Brett L. Walker, Linda Nash, “Forum: Technology, Ecology, and Human Health Since 1850,” *Environmental History* 20 (2015): 710–804.

13 Chris Otter, Nicholas Breyfogle, and John Brooke, “Introduction,” in ibid., 712.

14 Johnathan Patz, Peter Daszak, Gary M. Tabor, A. Alonso Aguirre, Mary Pearl, Jon Epstein, Nathan D. Wolfe, A. Marm Kilpatrick, Johannes Foufopoulos, David Molyneux, David J. Bradley, “Unhealthy Landscapes: Policy Recommendations on Land Use Change and Infectious Disease Emergence,” *Environmental Health Perspectives* 112 (2004):1092-1098.

15 Mari Webel, “Mapping the Infected Landscape: Sleeping Sickness Prevention and the African Production of Colonial Knowledge in the Early Twentieth Century,” in “Forum,” 731; Alexandre I.R. White, *Epidemic Orientalism: Race, Capital, and the Governance of Infectious Disease* (Stanford: Stanford University Press, 2023).

16 Chris Otter, “Toxic Foodways: Agro Food-Systems, Emerging Foodborne Pathogens, and Evolutionary History,” in “Forum”; Chris Otter, *Diet for a Large Planet: Industrial Britain, Food Systems, and World Ecology* (Chicago: University of Chicago Press, 2020); Guenter B. Risse, “Epidemics and History: Ecological Perspectives,” in *AIDS: The Burdens of History*, ed. Elizabeth Fee and Daniel M. Fox (Berkeley: University of California Press, 1988), 33-66.

17 For examples of evolution and ecology forming frames for environmental history (though less directly related to the question of disease), see Timothy J. LeCain, *The Matter of History: How Things Create the Past* (Cambridge: Cambridge University Press, 2017); Edmund Russell, *Evolutionary History: Uniting History and Biology to Understand Life on Earth* (Cambridge: Cambridge University Press, 2010); Daniel Lord Smail, *On Deep History and the Brain* (Berkeley: University of California Press, 2007).

18 F. John Odling-Smee, Kevin Laland, and Marcus Feldman, *Niche Construction: The Neglected Process in Evolution* (Princeton: Princeton University Press, 2003); Kevin Laland, Blake Matthews, and Marcus W. Feldman, “An Introduction to Niche Construction Theory,” *Evolutionary Ecology* 30 (2016): 191-202; Kevin N. Laland and Michael J. O’Brien, “Cultural Niche Construction: An Introduction,” *Biological Theory* 6 (2012): 191-202.

19 Emily Webster, “Tubercular Landscape: Land Use Change and Mycobacterium in Melbourne, Australia, 1837–1900,” *Journal of Historical Geography* 67 (2020): 48–60; J.R. McNeill, *Mosquito Empires: Ecology and War in the Greater Caribbean, 1620–1914* (Cambridge: Cambridge University Press, 2010); Andrew Price-Smith, “The Plagues of Affluence: Human Ecology and the Case of the SARS Epidemic,” in “Forum,” 765-778.

20 Dunk and Anderson, “Assembling Planetary Health,” 17-36; James H. Dunk, David S. Jones, Anthony Capon, Warwick H. Anderson, “Human Health on an Ailing Planet — Historical Perspectives on Our Future,” *New England Journal of Medicine* 381 (2019): 778-782.

21 Beyond the works cited in this piece, see Randall Packard, *The Making of a Tropical Disease: A Short History of Malaria* (Baltimore: Johns Hopkins University Press, 2007); Angela Cassidy, *Vermin, Victims, and Disease: British Debates over Bovine Tuberculosis and Badgers* (Cham: Palgrave MacMillan, 2019).

22 Monica H. Green, ed., *Pandemic Disease in the Medieval World: Rethinking the Black Death* (Kalamazoo, MI: Arc Humanities Press, 2015), 15.

23 David Quammen, *Spillover: Animal Infections and the Next Human Pandemic* (New York: Norton, 2013).

24 Google trends examined between 2004-2022 shows a pronounced peak in the use of terms “zoonosis,” “zoonotic,” and “spillover” in March 2020. “‘Zoonotic, Zoonoses, Spillover,’” Google trends, accessed 12 September 2022, https://trends.google.com/trends/.

25 Christos Lynteris and Lyle Fearnley, “Why Shutting down Chinese ‘Wet Markets’ Could Be a Terrible Mistake,” *EconoTimes*, 3 February 2020, https://www.econotimes.com/Why-shutting-down-Chinese-wet-markets-could-be-a-terrible-mistake-1573706.

26 Dolly Jorgensen, “Tracking Animals in a Pandemic,” *Environmental History* 25 (2020): 626-631.

27 Ibid.; Edmund Russell, *Greyhound Nation: A Coevolutionary History of England, 1200-1900* (Cambridge: Cambridge University Press, 2018); LeCain, *Matter of History*.

28 A.J. Hovorka, “Animal Geographies I: Globalizing and Decolonizing,” *Progress in Human Geography* 41 (2017): 382-394; Peter Atkins, *Animal Cities: Beastly Urban Histories* (London: Routledge Press, 2012); Andrew Robichaud, *Animal City: The Domestication of America* (Cambridge, MA: Harvard University Press, 2019); Philip Howell and Hilda Kean, “Writing in Animals in History,” in *The Routledge Companion to Animal-Human History*, ed. Hilda Kean and Philip Howell (London: Routledge Press, 2018), 3-27. On the historical contingency of human non-human relationships in the Anthropocene, see Dipesh Chakrabarty, *The Climate of History in a Planetary Age* (Chicago: University of Chicago Press, 2021), 126-127.

29 Daniel R. Headrick, “Sleeping Sickness Epidemics and Colonial Responses in East and Central Africa, 1900–1940,” *PLoS Neglected Tropical Diseases* 8 (2014): e2772; John McNeill, *Mosquito Empires: Ecology and War in the Greater Caribbean, 1620-1914* (Cambridge: Cambridge University Press, 2010); Dawn Biehler, *Pests in the City: Flies, Bedbugs, Cockroaches, and Rats* (Seattle: University of Washington Press, 2015); Urmi Engineer Willoughby, *Yellow Fever, Race, and Ecology in Nineteenth-Century New Orleans* (Baton Rouge: Louisiana State University Press, 2017).

30 Christos Lynteris, *Framing Animals as Epidemic Villains: Histories of Non-Human Disease Vectors* (Cham: Palgrave Macmillan, 2019); Christos Lynteris, “The Global War Against the Rat and the Epistemic Emergence of Zoonosis,” University of St. Andrews, 2020, https://wwrat.wp.st-andrews.ac.uk/; Green, *Pandemic Disease in the Medieval World*, 20.

31 Lynteris, *Framing Animals as Epidemic Villains*, 2.

32 Ann Hardy, *Salmonella Infections, Networks of Knowledge, and Public Health in Britain, 1880-1975* (Oxford: Oxford University Press, 2015).

33 For examples of work in this field, see Otter, *Diet for a Large Planet*; Peter Atkins, ed., *Animal Cities: Beastly Urban Histories* (New York: Taylor & Francis, 2012).

34 LeCain, *Matter of History*; Julia Adeney Thomas, “History and Biology in the Anthropocene: Problems of Scale, Problems of Value,” *American Historical Review* 119 (2014): 1587-1607.

35 Thomas, “History and Biology in the Anthropocene,” 1588.

36 Ibid.

37 For a more detailed discussion of the theorization of scale in geographic and spatial thinking, see Sallie A. Marston, John Paul Jones III, Keith Woodward, “Human Geography Without Scale,” *Transactions of the Institute of British Geographers* 30 (2004): 416-432.

38 Monica H. Green, “The Four Black Deaths,” *American Historical Review* 125 (2020): 1601-1631; Green, *Pandemic Disease in the Medieval World*; Michael McCormick, “Rats, Communications, and Plague: Towards an Ecological History,” *Journal of Interdisciplinary History* 34 (2003): 1-25; Stefan Monecke, Hannelore Monecke, and Jochen Monecke, “Modelling the Black Death: A Historical Case Study and Implications for the Epidemiology of Bubonic Plague,” *International Journal of Medical Microbiology* 299 (2009): 582-593; McNeill, *Plagues and Peoples*; Mark Harrison, *Contagion: How Commerce Has Spread Disease* (New Haven: Yale University Press, 2012); Mark Harrison, *Public Health in British India: Anglo-Indian Preventive Medicine, 1859-1914* (Cambridge: Cambridge University Press, 2008); Myron Echenberg, *Plague Ports: The Global Urban Impact of Plague 1894-1901* (New York: New York University Press, 2010); Christos Lynteris, “A ‘Suitable Soil’: Plague’s Urban Breeding Grounds at the Dawn of the Third Pandemic,” *Medical History* 61 (2017): 343-357.

39 See for example, Linda Nash, “Writing Histories of Disease and Environment in the Age of the Anthropocene,” *Environmental History* 20 (2015): 796-804.

40 John Bellamy Foster, Brett Clark, and Hannah Holleman, “Capitalism and the Ecology of Disease,” *Monthly Review*, 1 June 2021.

41 Gregg Mitman, “The Unruliness of a Virus,” in Peter Alagona, Jane Carruthers, Hao Chen, Michèle Dagenais, Sandro Dutra e Silva, Gerard Fitzgerald, Shen Hou, Dolly Jørgensen, Claudia Leal, John McNeill, Gregg Mitman, Gabriella Petrick, Liza Piper, Libby Robin, Edmund Russell, Christopher Sellers, Mart A Stewart, Frank Uekötter, Conevery Bolton Valencius, and Marco Armiero, “Reflections: Environmental History in the Era of COVID-19,” *Environmental History* 25 (2020): 641.

42 Ibid., 642.

43 Dipesh Chakrabarty, “The Climate of History, Four Theses,” *Critical Inquiry* 35 (2009): 197-222.

44 Monica H. Green, “Editor’s Introduction,” in *Pandemic Disease in the Medieval World*, 9-26; Alex Liebman and Robert G. Wallace, “A Factory Farm Fungus Among Us,” *The Feral Atlas*, accessed 15 August 2022, https://feralatlas.supdigital.org/poster/a-factory-farm-fungus-among-us; Michael G. Vann and Liz Clarke, *The Great Hanoi Rat Hunt: Empire, Disease, and Modernity in French Colonial Vietnam* (Oxford: Oxford University Press, 2019).

45 Nash, “Beyond Virgin Soil Epidemics,” 93.

46 Center for Systems Science and Engineering (CSSE) at Johns Hopkins University (JHU), “COVID-19 Dashboard,” *Johns Hopkins University and Medicine*, Accessed 28 September 2022, https://coronavirus.jhu.edu/map.html.

47 Munazza Fatima, Kara J. O’Keefe, Wenjia Wei, Sana Arshad, Oliver Gruebner, “Geospatial Analysis of COVID-19: A Scoping Review,” *International Journal of Environmental Research and Public Health* 18 (2021): 2336; Vida Abedi, Oluwaseyi Olulana, Venkatesh Avula, Durgesh Chaudhary, Ayesha Khan, Shima Shahjouei, Jiang Li, Ramin Zand, “Racial, Economic, and Health Inequality and COVID-19 Infection in the United States,” *Journal of Racial and Ethnic Health Disparities* 8 (2021): 732–742.

48 Michael Worobey, Joshua I. Levy, Lorena Malpica Serrano, Alexander Crits-Christoph, Jonathan E. Pekar, Stephen A. Goldstein, Angela L. Rasmussen, Moritz U.G. Kraemer, Chris Newman, Marion P.G. Koopmans, Marc A. Suchard, Joel O. Wertheim, Philippe Lemey, David L. Robertson, Robert F. Garry, Edward C. Holmes, Andrew Rambaut, and Kristian G. Andersen, “The Huanan Seafood Wholesale Market in Wuhan Was the Early Epicenter of the COVID-19 Pandemic,” *Science* 377 (2022): 951-959.

49 William T. Harvey, Alessandro M. Carabelli, Ben Jackson, Ravindra K. Gupta, Emma C. Thomson, Ewan M. Harrison, Catherine Ludden, Richard Reeve, Andrew Rambaut, COVID-19 Genomics UK Consortium, Sharon J. Peacock, David L. Robertson, “SARS-CoV-2 Variants, Spike Mutations and Immune Escape,” *Nature Reviews Microbiology* 19 (2021): 409–424.

50 Monica Green, “Taking ‘Pandemic’ Seriously: Making the Black Death Global,” in *Pandemic Disease in the Medieval World*, 29; Green, “Four Black Deaths,” 1605; Ann G. Carmichael, “Plague Persistence in Western Europe: A Hypothesis,” in *Pandemic Disease in the Medieval World*, 157-193.

51 Helen Bynum, *Spitting Blood: A History of Tuberculosis* (Oxford: Oxford University Press, 2012).

52 Ibid.

53 Christian McMillen, *Discovering Tuberculosis: A Global History, 1900 to the Present* (New Haven: Yale University Press, 2015), Introduction.

54 Geoffrey C. Bowker and Susan Leigh Starr, *Sorting Things Out: Classification and its Consequences* (Cambridge, MA: MIT Press, 2000), cited in Claas Kirchhelle and Charlotte Kirchhelle, “Northern Normal – Laboratory Networks, Microbial Culture Collections, and Taxonomies of Power (1939-2000),” *SocArXiv* (2022): doi:10.31235/osf.io/yv5ck.

55 To see propositions for a responsible use of climate models, see Dagomar Degroot, Kevin Anchukaitis, Martin Bauch, Jakob Burnham, Fred Carnegy, Jianxin Cui, Kathryn de Luna, Piotr Guzowski, George Hambrecht, Heli Huhtamaa, Adam Izdebski, Katrin Kleemann, Emma Moesswilde, Naresh Neupane, Timothy Newfield, Qing Pei, Elena Xoplaki, Natale Zappia, “Towards a Rigorous Understanding of Societal Responses to Climate Change,” *Nature* 591 (2021): 539-550. For an earlier example of such responsible use, see Mike Davis, *Late Victorian Holocausts: El Niño Famines and the Making of the Third World* (London: Verso, 2000).

56 J. Luterbacher, T.P. Newfield, E. Xoplaki, E. Nowatzki, N. Luther, M. Zhang, N. Khelifi, “Past Pandemics and Climate Variability across the Mediterranean,” *Euro-Mediterranean Journal for Environmental Integration* 5 (2020): 46.

57 Ibid.

58 Tom Koch, *Disease Maps: Epidemics on the Ground* (Chicago: University of Chicago Press, 2011); Jacob Steere-Williams, *The Filth Disease: Typhoid Fever and the Practices of Epidemiology in Victorian England* (Rochester: University of Rochester Press, 2020); Lukas Engelmann, *Mapping AIDS: Visual Histories of an Enduring Epidemic* (Cambridge: Cambridge University Press, 2018).

59 Koch, *Disease Maps*, 2.

60 Ibid., 12-14.

61 Engelmann, *Mapping AIDS*; Koch, *Disease Maps*.

62 Koch, *Disease Maps*, 5.

63 Jacques Pépin, *The Origins of AIDS*, 2nd ed. (Cambridge: Cambridge University Press, 2021). For other examples of historical disease mapping, see Fabian Crespo and Matthew B. Lawrenz, “Heterogeneous Immunological Landscapes and Medieval Plague: An Invitation to a New Dialogue Between Historians and Immunologists,” in *Pandemic Disease and the Medieval World*, 241-243; Ann Carmichael, “Plague Persistence in Western Europe: A Hypothesis,” in *Pandemic Disease in the Medieval World*, 163; Stuart Borsch, “Plague Depopulation and Irrigation Decay in Medieval Egypt,” in *Pandemic Disease in the Medieval World*, 131-149; Green, “Four Black Deaths.”

64 Steere-Williams, *Filth Disease*; Hardy, *Salmonella Infections, Networks of Knowledge, and Public Health in Britain*; John Brewer, *Sinews of Power: War Money and the English State: 1688-1783* (Cambridge, MA: Harvard University Press, 1990).

65 Michel Foucault, *The Birth of Biopolitics: Lectures at the College of France 1978-1979* (New York: MacMillan Press, 2010).

66 Engelmann, *Mapping AIDS*, 103.

67 Ibid., 141.

68 Typhoid, Cockles, and Terrorism is an AHRC-IRC Digital Humanities Project that explores the impact and limits of imperial sanitary and microbiological interventions in the turbulent context of colonial and revolutionary Ireland. For more information, see Claas Kirchhelle, Sam Vanderslott, Carly Collier, and Emily Webster, “Typhoidland,” 12 March 2024, https://typhoidland.org/.

69 Charles Cameron, “On Sewage in Oysters, presented at the Forty-Eighth Annual Meeting of the British Medical Association,” *British Medical Journal* 2 (1880): 465-480.

70
*Report of the Committee Appointed by the Local Government Board for Ireland to Inquire into the Public Health of the City of Dublin* (Dublin: Alexander Thom. & Co., 1900), House of Commons Parliamentary Papers Online, 38.

71 Steere-Williams, *Filth Disease*, 77-127.

72 T.W. Grimshaw and Sir Charles A. Cameron, “Appendix. The Distribution of Enteric Fever in the City of Dublin,” in Cameron, *Report upon the State of Public Health and the Sanitary Work Performed in Dublin During the Year 1888: Also, Twenty-seventh Annual Report Upon the Analysis and Inspection of Food &c. by Sir Charles A. Cameron, MD* (Dublin: Falconer, 1889), Reports and Printed Documents of the Corporation of Dublin, 1889, vol.2, Dublin City Library and Archives, Dublin, Ireland.

73
*Report upon the State of Public Health and the Sanitary Work Performed in Dublin During the Year 1880; Also, Nineteenth Annual Report Upon the Analysis and Inspection of Food, &c. by Charles A. Cameron, MD* (Dublin: Dollard, 1881), Reports and Printed Documents of the Corporation of Dublin, 1881, vol.2. DCLA.

74 Jonathan A. Polonsky, Isabel Martinez-Pino, Fabienne Nackers, Prosper Chonzi, Portia Manangazira, Michel Van Herp, Peter Maes, Klaudia Porten, Francisco J. Luquero, “Descriptive Epidemiology of Typhoid Fever During an Epidemic in Harare, Zimbabwe, 2012,” *PLoS One* 9 (2014): e114702; Aaron P. Jenkins, Stacy D. Jupiter, Adam Jenney, Varanisese Rosa, Alanieta Naucukidi, Namrata Prasad, Gandercillar Vosaki, Kim Mulholland, Richard Strugnell, Mike Kama, John A. Crump, Pierre Horwitz, “Environmental Foundations of Typhoid Fever in the Fijian Residential Setting,” *International Journal of Environmental Research and Public Health* 16 (2019): 2407-2427.

75 Aaron Jenkins, “A Nested Environmental Approach to Typhoid Epidemiology in Central Division, Fiji” (PhD diss., Edith Cowan University, 1992).

76 Ibid., 9.

77 Aaron Peter Jenkins, Stacy Jupiter, Ute Mueller, Adam Jenney, Gandercillar Vosaki, Varanisese Rosa, Alanieta Naucukidi, Kim Mulholland, Richard Strugnell, Mike Kama, Pierre Horwitz, “Health at the Sub-catchment Scale: Typhoid and Its Environmental Determinants in Central Division, Fiji,” *EcoHealth* 13 (2016): 633-651, 644.

78 Elizabeth Gosling, *Bivalve Molluscs: Biology, Ecology and Culture* (Oxford: Blackwell Press, 2003), 4-5.

79 D. Edgar Flinn, “Report on the Sanitary State of Dublin,” “Enclosed – Official Report on the Sanitary Circumstances and Administration of the City of Dublin, with Special Reference to the Causes of the High Death-Rate,” 1906, CSO/RP/IR/5294, General Administration, National Archives of Ireland.

80 Ibid., 29.

81 Royal Sanitary Commissioners, Dublin, Report of the Royal Commissioners Appointed to Inquire into the Sewerage and Drainage of the City of Dublin and Other Matters Connected Therewith, together with Minutes of Evidence, Appendix, Index, &c. WA785 1880G784, Wellcome Collection, London, United Kingdom.

82 Ibid., 114.

83 Charles Cameron, “Report upon the State of Public Health and the Sanitary Work Performed in Dublin During the Year 1880; Also, Nineteenth Annual Report Upon the Analysis and Inspection of Food, &c. by Charles A. Cameron, MD,” in *Dublin Corporation Reports &c for the Year 1881*, vol. 2, 71, DCLA.

84 Charles Cameron, “Report upon the State of Public Health and the Sanitary Work Performed in Dublin during the Year 1889,” in *Dublin Corporation Reports &c for the Year 1889*, vol. 2, 620.

85 Cameron, “Report on the State of Public Health in the City of Dublin for the Year 1903,” in *Dublin Corporation Reports &c for the Year 1903*, vol. 2, 71, DCLA.

86 Ibid.

87 “Woman Dies After Eating Cockles,” *Irish Times*, 10 August 1946.

88 Nash, “Beyond Virgin Soil Epidemics,” 86.

